# A Qualitative Transcriptional Signature for Predicting Recurrence Risk of Stage I–III Bladder Cancer Patients After Surgical Resection

**DOI:** 10.3389/fonc.2019.00629

**Published:** 2019-07-10

**Authors:** Yawei Li, Huarong Zhang, You Guo, Hao Cai, Xiangyu Li, Jun He, Hung-Ming Lai, Qingzhou Guan, Xianlong Wang, Zheng Guo

**Affiliations:** ^1^Key Laboratory of Ministry of Education for Gastrointestinal Cancer, Department of Bioinformatics, School of Basic Medical Sciences, Fujian Medical University, Fuzhou, China; ^2^Medical Big Data and Bioinformatics Research Centre, First Affiliated Hospital of Gannan Medical University, Ganzhou, China; ^3^Key Laboratory of Medical Bioinformatics, Fujian Medical University, Fuzhou, China

**Keywords:** bladder cancer, micro-metastasis, qualitative transcriptional signature, differentially expressed genes, differentially methylated genes

## Abstract

**Background:** Previously reported transcriptional signatures for predicting the prognosis of stage I-III bladder cancer (BLCA) patients after surgical resection are commonly based on risk scores summarized from quantitative measurements of gene expression levels, which are highly sensitive to the measurement variation and sample quality and thus hardly applicable under clinical settings. It is necessary to develop a signature which can robustly predict recurrence risk of BLCA patients after surgical resection.

**Methods:** The signature is developed based on the within-sample relative expression orderings (REOs) of genes, which are qualitative transcriptional characteristics of the samples.

**Results:** A signature consisting of 12 gene pairs (12-GPS) was identified in training data with 158 samples. In the first validation dataset with 114 samples, the low-risk group of 54 patients had a significantly better overall survival than the high-risk group of 60 patients (HR = 3.59, 95% CI: 1.34~9.62, *p* = 6.61 × 10^−03^). The signature was also validated in the second validation dataset with 57 samples (HR = 2.75 × 10^08^, 95% CI: 0~Inf, *p* = 0.05). Comparison analysis showed that the transcriptional differences between the low- and high-risk groups were highly reproducible and significantly concordant with DNA methylation differences between the two groups.

**Conclusions:** The 12-GPS signature can robustly predict the recurrence risk of stage I-III BLCA patients after surgical resection. It can also aid the identification of reproducible transcriptional and epigenomic features characterizing BLCA metastasis.

## Introduction

Bladder cancer (BLCA) is still a major health problem worldwide (1). For stage I-III BLCA patients without metastasis, transurethral resection of bladder tumor (stage I) or radical cystectomy (stage II–III) is the standard treatment, which can improve patients' survival. However, a study based on large samples reported that, after radical cystectomy, the 5 years mortality rate for stage I patients and stage II-III patients are only about 18.4 and 36.2%, respectively (2, 3), leading to the poor prognosis of BLCA patients (4). An important reason is that occult micro-metastases contribute to high recurrence risk of patients (5). Currently, both imaging techniques (6–8) and pathological evaluation techniques even with an enlarging pathological evaluation (9) have relatively high false-negative rates for occult micro-metastases, which are likely responsible for most of the recurrence or mortality (6–8). Therefore, it is urgent to develop an accurate molecular signature for predicting potential micro-metastasis states of postoperative patients.

Many transcriptional signatures for predicting prognosis or recurrence risk of BLCA patients after curative surgery have been provided (10–13). However, a critical limitation of such quantitative signatures is that their applications are commonly based on risk scores calculated as quantitative summaries of the expression measurements of the signature genes, which are impractical for clinical applications due to large measurement batch effects (14, 15) and quality uncertainties of clinical samples (16–19). In contrast, the signatures based on the within-sample relative expression orderings (REOs) of genes are robust against experimental batch effects (20) and can be applied to individual disease samples assessed in different laboratories (14, 21–24).Besides, the REO-based signatures are highly robust against varied proportions of the tumor epithelial cell in tumor tissues sampled from different tumor locations of the same patient (18), partial RNA degradation during specimen storage and preparation (17) and amplification bias for minimum specimens even with about 15–25 cancer cells (19), which are common factors leading to failures of quantitative transcriptional signatures in clinical applications. Therefore, it is feasible to identify a REOs-based signature for predicting recurrence risk of stage I–III BLCA patients after surgical resection.

In this study, a REOs-based prognostic signature, composed of 12 gene pairs, was identified in training dataset, and this signature was validated in two independent datasets. Then, we used the BLCA samples from The Cancer Genome Atlas (TCGA), to analyze the genomic and epigenomic gene markers characterizing the two prognostic groups.

## Materials and Methods

### Data Sources and Data Preprocessing

The TCGA data, measured by the Illumina-Hiseq platform, was downloaded from TCGA data portal website (http://cancergenome.nih.gov/). From the TCGA RNA-seq data, we extracted 158 samples of patients who did not receive chemotherapy, neoadjuvant or radiotherapy, denoted as BLCA158, for training the signature. The mRNA-seq profiles of level 3 Fragments Per Kilobase Million (FPKM) was extracted as the gene expression measurements for the dataset BLCA158. Another two datasets of BLCA samples profiled by different microarray platforms were downloaded from the Gene Expression Omnibus (GEO, http://www.ncbi.nlm.nih.gov/geo/). From the GSE31684 dataset measured by the Affymetrix Human Genome U133 Plus 2.0 Array (GPL570), 57 samples of patients treated with curative surgery alone were extracted and used as the first validation dataset, denoted as BLCA57. From the GSE32894 dataset measured by the Illumina HumanHT platform GPL6974, we extracted 114 samples of stage I-III patients treated with curative surgery alone, excluding samples of patients treated with radiotherapy, as the second validation dataset, denoted as BLCA114. Data description is summarized in Table 1. Probe-set IDs were mapped to gene IDs using the corresponding platform files. If multiple probesets were mapped to the same gene, the expression value of the gene was summarized as the arithmetic mean of the values of the multiple probesets for the GSE31684 and GSE32894 datasets.

**Table 1:** Description of the datasets used in this study.

		**Training datasets**	**Validation datasets**
		BLCA158	BLCA57	BLCA114
		TCGA	GSE31684	GSE32894
Platform		Illumina Hiseq-RNAseq	GPL570	GPL6974
Sample Size		158	57	114
Stage	0	0	5	0
	I	1	9	63
	II	60	12	43
	III	58	22	8
	IV	39	9	0

For the 158 samples of BLCA158 dataset, the DNA methylation beta-values of samples for 15,932 genes measured by the Infinium HumanMethylatio450 platform was derived from the TCGA Web Portal.

### Survival Analysis

The disease-free survival (DFS) was defined as the time from surgical resection to the date of tumor recurrence or distant metastasis, and overall survival (OS) was defined as the time from surgery to death. Survival curves of DFS and OS between distinct subgroups were estimated by the Kaplan-Meier method and compared with log-rank tests (25). The univariate Cox regression model was used to evaluate the correlation of expression values of genes and the REOs of gene pairs with patients' OS. The multivariate Cox regression model was used to evaluate the independent prognostic value of the signature after adjusting for clinical features including age, gender, and stage, which could be written as follow:

$$\begin{array}{l} h\left(t,x\right)=h_{0}\left(t\right)\exp\left(\beta_{1}x_{1}+\beta_{2}x_{2}+\;\cdots \;+\;\beta_{n}x_{n}\right) \end{array}$$

where *h*(*t, x*) is the hazard function determined by a set of *n* covariates (*x*_1_, *x*_2_ … *x*_*n*_)and *t*refers to the survival time, the coefficients (β_1_, β_2_ … β_*n*_) measure the impact (i.e., the effect size) of covariates. *h*_0_ describes the baseline hazard. It corresponds to the value of the hazard if all the *x*_*i*_ is equal to zero.

The C-index is calculated as the proportion of consistent outcomes among all possible high-low risk sample pairs (26), which takes values ranging from 0.5 to 1, where 0.5 and 1 represent the worst and best prediction ability, respectively.

### Identification of Prognostic Gene Pair Signature

For a gene pair, gene A and gene B, let E_A_ and E_B_ represent the expression measurements of gene A and gene B, respectively. And all samples were classified into two groups based on the pattern of REO (E_A_ > E_B_ or E_A_ < E_B_) in each sample. Using the gene expression of training dataset, we identified a gene pair signature. The detailed process is as follows:

1. The genes whose expression levels are significantly associated with patients' prognoses are identified by using the univariate Cox regression model.
2. Then, for each of the gene pairs, formed from every two of the genes selected above, all samples in the training dataset are divided into two groups according to the REO pattern of the gene pair in each of the samples. If the OS of the two groups of samples are significantly different (univariate Cox regression model), then the REO patterns of the gene pair are considered to be significantly associated with OS, denoted as the pre-selected candidate signature gene pairs. The *p*-values are adjusted using the Benjamini–Hochberg (BH) procedure (27).
3. From the pre-selected candidate signature gene pairs, the gene pair with the highest C-index value is chosen as the seed signature, and the candidate signature gene pairs are added to the signature one at a time, according to their C-index values ranked in descending order, until the addition of any gene pair does not improve the C-index.
4. A forward selection procedure is performed to search an optimal subset of the candidate signature gene pairs that achieves the highest C-index based on the pre-defined classification rule: a patient is classified into the high-risk group if at least half of the gene pairs of this patient vote for high risk; otherwise, the low-risk group.
5. Finally, the subset of gene pairs with the highest C-index was chosen as the final prognostic gene pair signature.

### Analysis of Transcriptional, Epigenomic, and Genomic Data

The Significance Analysis of Microarrays (SAM) method was used to select differentially expressed genes (DEGs) between two groups of samples from BLCA57 and BLCA114 datasets, respectively. Because the RankCompV2 algorithm for detecting DEGs is insensitive to batch effects (28), we use this algorithm to analyze samples from the dataset BLCA158 spread over 15 batches.

The Wilcoxon rank-sum test was used to identify differentially methylated genes (DMGs) between the low- and high-risk samples. Fisher's exact test was used to detect copy number alternations or gene mutations which have significantly different frequencies between two prognostic groups.

### Concordance Scores

A concordance score was defined to evaluate the consistency between two lists of DEGs separately detected from two independent datasets. For the two lists of DEGs, if there are *k* overlapping DEGs, of which *s* genes showed the same dysregulation directions (overexpressed or underexpressed), the concordance score was calculated as the ratio, *s*/*k*. If *k* genes were both hypermethylated (or hypomethylated) and differentially expressed, of which *s* genes were correspondingly underexpressed (or overexpressed), the concordance score was calculated as *s*/*k*. This score was used to evaluate the concordance between DEGs and DMGs. The probability of observing a concordance score of *s*/*k* by chance is evaluated by the cumulative binomial distribution model (29):

$$P=1-\overset{s-1}{\underset{i=0}{\sum }}\left(\frac{k}{i}\right){\left(P_{0}\right)}^{i}{\left(1-P_{0}\right)}^{k-i}\;$$

where *p*_0_ (here, *p*_0_ = 0.5) is the probability of a gene having the concordant relationship between the two gene lists by random chance.

### Pathway Enrichment Analysis

GoFunction algorithm was performed to select GO biological pathways that significantly enriched with DEGs. The BH procedure (27) was used to calculate the False Discovery Rate (FDR). All statistical analyses were done by using the R software package version 3.1.3.

## Results

### Development of the REOs-Based Prognostic Signature

Using the 158 BLCA samples with no drug treatment from the BLCA158 dataset measured by Illumina-Hiseq, we identified 76 genes whose expression values were significantly correlated with the OS of the 158 BLCA patients (univariate Cox proportional-hazards regression model, FDR <5%). For all gene pairs combined by the 76 genes, 521 OS-associated gene pairs were selected according to their REOs in each sample (univariate Cox regression model, FDR <1%). For each of the 521 pre-selected candidate signature gene pairs, we classified each of the 158 samples from the BLCA158 dataset into the low- or high-risk group according to the REO of the gene pair in this sample and calculated the C-index value to evaluate the prognostic prediction power of the gene pair (see section Materials and Methods). Then, with the forward selection procedure described in section Materials and Methods, we identified a set of 12 gene pairs that reached the highest C-index value (C-index = 0.80) with the majority voting rule (see section Materials and Methods). The 12 gene pairs, denoted as 12-GPS (Table 2), classified patients into the high-risk group if at least six gene pairs vote for high risk; otherwise, the low-risk group (see section Materials and Methods).

**Table 2:** The 12-GPS prognostic signature.

**Pair**	**Gene A**	**Gene B**	**Beta**	**C-index**
Pair1	*CRAMP1L*	*BAIAP2*	1.21	0.68
Pair2	*TALDO1*	*EMP1*	1.40	0.65
Pair3	*ALG3*	*TSPAN7*	1.22	0.64
Pair4	*ANGEL2*	*DDX10*	1.31	0.64
Pair5	*PSMG1*	*SLC7A11*	2.38	0.61
Pair6	*CRAMP1L*	*DDX10*	2.07	0.59
Pair7	*PRPF4B*	*UTP6*	1.78	0.58
Pair8	*TIA1*	*PSMD11*	1.28	0.57
Pair9	*ALYREF*	*ALG3*	1.18	0.54
Pair10	*PDPK1*	*ANGPT1*	1.3	0.54
Pair11	*PSD4*	*POPDC3*	1.67	0.53
Pair12	*KMT2B*	*PSMG1*	1.97	0.53

*Beta calculated by the univariate Cox regression model represents the risk coefficient of within-sample REO of gene pair (A, B), where Beta > 0 indicates that E_A_ < E_B_ is a risk factor*.

**Figure 1:**
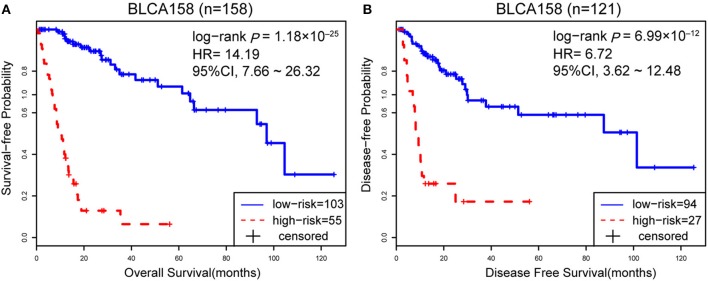
The Kaplan-Meier curves of DFS and OS for prognostic groups predicted by 12-GPS in the training dataset. A patient was classified into the high-risk group when more than half of the gene pairs in the 12-GPS vote for high risk, and vice versa. Kaplan-Meier curves of OS for the training dataset BLCA158 **(A)**; Kaplan-Meier curves of DFS for the training dataset BLCA158 **(B)**.

**Figure 2:**
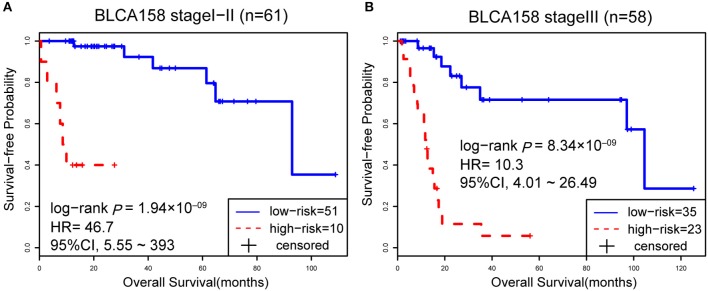
The Kaplan-Meier curves of OS for 61 stage I-II **(A)** and 58 stage III **(B)** BLCA patients from training dataset BLCA158.

Accordingly, patients in the BLCA158 dataset were classified into the low-risk (103 samples) and high-risk (55 samples) groups, which the OS of the former group is significantly better than the latter group (HR = 14.19, 95% CI: 7.66~26.32, *p* = 1.18 × 10^−25^, Figure 1A). In the 121 samples from the BLCA158 dataset, the DFS of low-risk group (94 samples) is significantly better than the high-risk group (27 samples) (HR = 6.72, 95% CI: 3.62~12.48, *p* = 6.99 × 10^−12^, Figure 1B). Especially, the 12-GPS classified 51 and 10 patients of the 61 stage I-II patients into the low- and high-risk groups, respectively, and the OS of the former group is significantly increased compared to the latter group (HR = 46.7, 95% CI: 5.55~393, *p* = 1.94 × 10^−09^, Figure 2A). The signature could also classify the 58 stage III samples into the low-risk group (35 samples) and the high-risk group (23 samples) with a significantly different OS (HR = 10.30, 95% CI: 4.08~26.49, *p* = 8.34 × 10^−09^, Figure 2B). Besides, the 12-GPS remained significantly associated with patients' OS after adjusting for AJCC stage, grade, gender and age (multivariate Cox proportional-hazards regression model, HR = 11.26, 95% CI: 5.68~22.32, *p* = 3.89 × 10^−12^, Table 3).

**Table 3:** Univariate and multivariate Cox regression analyses for the 12-GPS signature.

**Variables**	**Univariate model**		**Multivariate model**	
	**HR (95% CI)**	***p***	**HR (95% CI)**	***p***
**BLCA158**				
Predictive signature (high vs. low)	14.09 (7.60 ~ 26.14)	2.00 × 10^−16^	11.89 (6.13 ~ 23.09)	2.60 × 10^−13^
Stage (I vs. II vs. III vs. IV)	2.40 (1.73 ~ 3.32)	3.00 × 10^−08^	1.66 (1.14 ~ 2.41)	0.0081
Gender (male vs. female)	0.73 (0.44 ~ 1.23)	0.20	0.76 (0.46 ~ 1.28)	0.31
Age	1.05 (1.02 ~ 1.07)	8.00 × 10^−04^	1.05 (1.01 ~ 1.08)	0.0042
Grade (high vs. low)	2.91 (0.40 ~ 21.17)	0.30	0.19 (0.02 ~ 1.63)	0.13
**The unified data of BLCA54 and BLCA114**				
Predictive signature (high vs. low)	4.31 (1.32 ~ 14.11)	0.0085	3.39 (1.01 ~ 11.39)	0.049
Stage (I vs. II vs. III vs. IV)	2.12 (1.46 ~ 3.07)	4.92 × 10^−05^	2.02 (1.35 ~ 3.01)	5.79 × 10^−04^
Age	0.98 (0.94 ~ 1.02)	0.36	0.99 (0.97 ~ 1.01)	0.20
Gender (male vs. female)	1.44 (0.60 ~ 3.49)	0.41	1.32 (0.54 ~ 3.21)	0.54
Grade (high vs. low)	2.00 (0.27 ~ 14.67)	0.49	0.61 (0.07 ~ 5.00)	0.64

### Validation of the Signature

**Figure 3:**
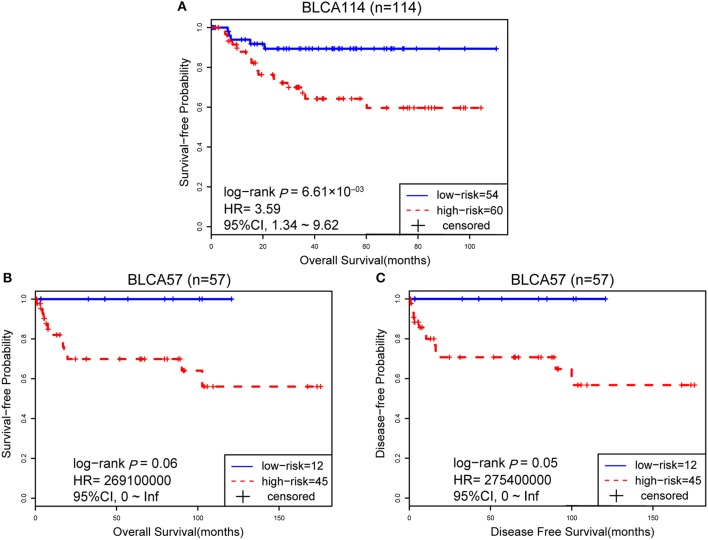
The performance of the 12-GPS for predicting OS in dataset BLCA114 **(A)**; Kaplan-Meier curves of OS **(B)**; and DFS **(C)** for the validation dataset BLCA57.

The 114 samples from the BLCA114 dataset were used as the first validation dataset. The signature classified 54 and 60 patients into the low- and high-risk groups, respectively. The low-risk group had a prolonged OS as compared to the high-risk group (HR = 3.59, 95% CI: 1.34~9.62, *p* = 6.61 × 10^−03^, Figure 3A). The second validation dataset was composed of 57 samples of patients with surgical alone from the BLCA57 dataset. Using the 12-GPS, 12 and 45 samples were classified into the low- and high-risk groups with a marginally significantly different OS but extremely high HR (HR = 2.69 × 10^08^, 95% CI: 0~Inf, *p* = 0.06, Figure 3B) mainly due to the small sample size. For the 57 samples with DFS data in this dataset, the low-risk group (12 samples) have a significantly better DFS than the high-risk group (45 samples) (HR = 2.75 × 10^08^, 95% CI: 0~Inf, *p* = 0.05, Figure 3C). Especially, the 12 patients of the low-risk group had no death or recurrence during the follow-up period.

**Figure 4:**
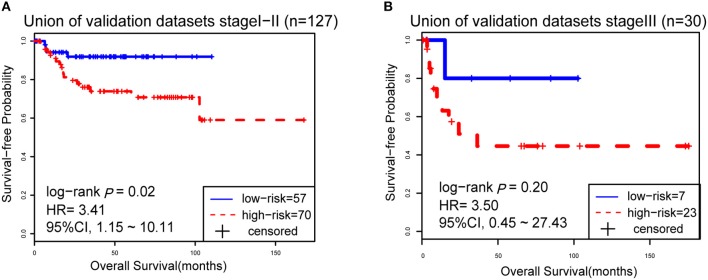
The Kaplan-Meier curves of OS for 127 stage I-II BLCA patients **(A)** and 30 stage III patients **(B)** from the unified of datasets BLCA114 and BLCA57.

**Figure 5:**
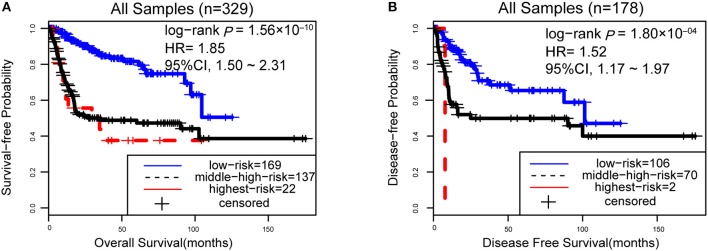
The performance of the 12-GPS for predicting OS **(A)** in the combined three datasets and DFS **(B)** in the combination of the datasets BLCA158 and BLCA57.

However, due to the lack of DFS information for the patients from the dataset BLCA114, we were only able to analyze the OS for the union of the dataset BLCA57 and BLCA114. For the combined data of the two validation datasets, the 12-GPS classified 57 stage I-II patients into the low-risk group and 70 stage I-II patients into the high-risk group, with significantly different OS (HR = 3.41, 95% CI: 1.15~10.11, *p* = 0.02, Figure 4A). And the OS of the low-risk patients of stage III stratified by the 12-GPS remained better than that of the high-risk patients although the difference was not significant which would be due to the limited number (*n* = 30) of samples in this dataset (HR = 3.50, 95% CI: 0.045~27.43, *p* = 0.20, Figure 4B). The biomarker proposed in this study is to predict the recurrence risk of patients only treated with curative surgery. And, this signature was developed by assuming that the existence of occult micro-metastases would cause stage I to III patients to have a relapse after curative surgery. That is, 12-GPS might not entirely be suitable for stage IV bladder cancer. In the combined data of BLCA158 and BLCA57, 48 stage IV patients were available and among them, 4 patients had distant metastases. As expected, we found that the developed signature was able to classify the four distantly metastatic patients into the high-risk group. The stratification of the other 48 stage IV patients was also statistically significant (HR = 6.53, 95% CI: 2.20~19.42, *p* = 1.32 × 10^−04^, Figure S1). Although 12-GPS was not initially designed for stage IV bladder cancer, it could predict the prognosis of these even more aggressive patients to some extent. Besides, within the high-risk group, the samples were further classified into the highest-risk group (Count _(EA < EB)_ ≥ 9, Table 2) if at least 9 gene pairs of 12-GPS vote for high risk, which had the poorer prognosis than those classified into the middle-high-risk group (9> Count _(EA < EB)_ ≥ 6, Table 2). Survival analysis for the integrated data of the three datasets showed that, as the threshold of gene pair number for supporting high risk increased to 9, the predicted highest-risk patients had even worse OS than the middle-high- and low-risk patients (HR = 1.86, 95% CI: 1.50~2.31, *p* = 1.56 × 10^−10^, Figure 5A). Similarly, in the combination of the datasets BLCA158 and BLCA57, the patients with the highest-risk had shorter DFS than the middle-high- and low-risk patients (HR = 1.52, 95% CI: 1.17~1.97, *p* = 1.80 × 10^−04^, Figure 5B). In the dataset BLCA57, the grade (reflecting the degree of tumor invasion) of a patient was evaluated as grade 1, grade 2 or grade 3, whereas in the dataset BLCA114 the grade of a patient was evaluated as high grade or low grade. Here, according to the Malignancy Grading of Bladder Carcinoma: Old and New Systems in NCCN Guidelines Version 1.2017 Bladder Cancer, in the unified dataset of BLCA 57 and BLCA114, we defined grade 1 as the low grade, and grade 3 as the high grade, excluding grade 2 samples from the analysis to avoid confusion. After adjusting for the AJCC stage, grade, age and gender, the 12-GPS remained significantly associated with patients' OS (multivariate Cox proportional-hazards regression model, HR = 3.39, 95% CI: 1.01~11.39, *p* = 0.049, Table 3) in the integrated data.

### Distinct Transcriptional and Epigenomic Characteristics of the Prognostic Subtypes

Between the stage IV (N+, M+ or T4b) samples and stage I-III (T1-T4aN0M0) samples from the BLCA114 and BLCA57 datasets, no DEG was identified (SAM, FDR <1%). Between the stage IV samples and stage I-III samples from the BLCA158 dataset, 73 DEGs were identified using the RankCompV2 algorithm (FDR <1%).Thus, the signals of differential expression between the primary tumors with metastasis and non-metastasis based on AJCC stage alone are weak and irreproducible in independent datasets, which is due to the high false positive and false negative reports of the commonly used imaging techniques and pathological examinations for tumor metastasis (6).

In contrast, we identified 2,171 and 2,356 DEGs (SAM, FDR <1%) between the low- and high-risk groups from the samples of the BLCA114 and BLCA57 datasets, respectively. Similarly, 922 DEGs were identified from the dataset BLCA158 using the RankCompV2 algorithm (FDR <1%). The consistency of DEGs between every two datasets is higher than 99.63% (binomial distribution test, *p* < 2.2 × 10^−16^, Table 4), suggesting significantly consistent transcriptional differences between the low- and high-risk groups.

**Table 4:** Concordance scores between DEGs detected from different datasets.

**Comparisons**	**Overlap**	**Consistence**	**Concordance scores**	***p-*value**
BLCA158 vs. BLCA114	475	475	100%	<2.20 × 10^−16^
BLCA158 vs. BLCA57	350	350	100%	<2.20 × 10^−16^
BLCA114 vs. BLCA57	800	797	99.63%	<2.20 × 10^−16^

By pooling the DEGs detected in the three datasets, we obtained 4,061 unique DEGs when excluding three DEGs with contradictory dysregulation directions between two datasets. Functional enrichment analysis showed that the 4,061 DEGs were significantly enriched in some pathways typically related to tumor metastasis such as “cell adhesion” (30), “cell migration” (30, 31), “cell differentiation” (32), and “cell division” (33) (FDR <1%, hypergeometric distribution model, Table S1).

According to the reclassified samples in BLCA158 dataset by 12-GPS, 605 hypermethylated and 736 hypomethylated genes were identified by comparing the methylation profiles of BLCA158 dataset between the two prognostic groups (Wilcoxon rank-sum test, FDR <1%), respectively. Two hundred and three genes out of the 605 hypermethylated genes were also overlapped with 4,061 DEGs, among which 94.09% were concordantly underexpressed in the high-risk group compared with the low-risk group, which was unlikely to happen by chance (binomial distribution test, *p* < 2.2 × 10^−16^). Similarly, we found that there are 284 overlaps between the 736 hypomethylated genes and the DEGs, among which 94.72% were concordantly overexpressed in the high-risk group, which was also highly unlikely to happen by chance (binomial distribution test, *p* < 2.2 × 10^−16^). Notably, using Fisher's exact test with FDR <1%, we were unable to detect genes which had significantly different copy number alternation frequencies or mutation frequencies between the two prognostic groups.

## Discussion

In this study, we developed a qualitative transcriptional signature based on the within-sample REOs of 12-GPS for predicting the DFS and OS of stage I-III BLCA patients after surgical resection. As mentioned in the Introduction, this REOs-based qualitative signature is highly robust against experimental batch effects, data normalization, varied proportions of the tumor epithelial cell in tumor tissues sampled from different tumor locations of the same patient (18), partial RNA degradation during specimen storage and preparation (17) and amplification bias for minimum specimens even with about 15–25 cancer cells (19). Therefore, it can be robustly applied at the individual level to samples measured by different laboratories. Remarkably, if treated with curative surgery only, the patients with low recurrence risk, classified by the signature, could not benefit from adjuvant therapy. After excluding these patients with adjuvant therapy-irrelevant low recurrence risk and collecting enough samples of patients treated with a particular neoadjuvant or adjuvant therapy, we can establish a predictive signature for the patients of high-risk group to identify which patients would response to a particular neoadjuvant or adjuvant therapy (34, 35).

For the establishment of predictive signatures for stage I-III bladder cancer patients who would be treated with curative surgery, we need to firstly identify the patients with adjuvant therapy-irrelevant low recurrence risk after surgery and exclude them from the analysis for predictive signatures because they would confound the identification of predictive signatures of response to a specific adjuvant therapy (34, 36). Therefore, the identification of prognostic signature for stage I-III bladder cancer patients treated with curative surgery only should be a basis for the establishment of predictive signatures of response to a specific adjuvant therapy. Next, we will develop a predictive signature to identify which patients could response to or benefit from a specific adjuvant therapy including chemotherapy or radiotherapy after collecting enough data of patients treated with a specific adjuvant therapy.

This qualitative signature can also help to determine the metastasis status under clinical settings based on the hypothesis that occult micro-metastases contribute to high recurrence risk of patients after surgical resection (5). Besides, we found several signature genes have important biological meaning associated with the carcinogenesis. For instance, TALDO1, as a nearly ubiquitous enzyme, has been linked to the progression of bladder cancer (37). Another signature gene, ALG3, has a higher expression in samples of breast cancer with advanced stages than those with early stages (38) and the decreased expression of PSD4 (EFA6B) was associated with poor prognosis of patients with breast cancer (39). Through the regulation of cellular invasiveness and migration, ALYREF is linked to local lymph node metastasis in human oral squamous cell carcinoma (40). Further, with the aid of 12-GPS, the tumor samples in the low- and high-risk groups showed a significantly consistent transcriptional differences characteristics related to tumor metastasis in independent datasets. Functional enrichment analysis showed that these transcriptional differences were significantly enriched in some classical metastasis-associated pathways, including “cell adhesion” (30), “cell migration” (30, 31), and “cell differentiation” (32). Further, we found that metastasis-associated DNA methylation alterations were significantly concordant with transcriptional differences observed between the low- and high-risk groups, indicating that DNA methylation alternations of the CpG loci in these genes play important roles in promoting cancer metastasis.

There are more than 60% of non-muscle invasive patients would recur and ~50% muscle invasive patients would develop metastases after curative surgery (2, 41, 42). It would be reasonable to assume that a major common factor, the existence of occult micro-metastases, leading to recurrence after curative surgery for the patients with the stage of localized/locally advanced, which would influence the choices of subsequent treatment for the bladder cancer patients. So, although the management is usually different between non-muscle invasive and muscle invasive cancer, we pooled them together into our analysis, while the later (muscle invasive cancer) would have higher probabilities of harboring occult micro-metastases and thus provide additional information for the discovery and validation of the signature for predicting the prognosis or recurrence risk (potential micro-metastasis) of patients after curative surgery (43).

In summary, given that the subtle quantitative measurements of gene expression are unreliable (15), the apparent shortcoming of this qualitative signature that it might lose some subtle quantitative information of gene expression is actually a unique merit of robustness. Therefore, it is worthy to be further verified in clinical practice.

## Conclusions

The REO-based 12-GPS prognostic signature is a true individual-level qualitative signature, which can robustly identify the stage I-III BLCA patients with potential occult micro-metastases and be an auxiliary tool for clinicians to determine whether patients should receive adjuvant therapy after surgical resection. Compared with the low-risk samples, the high-risk samples identified by 12-GPS have distinct transcriptional and epigenetic features characterizing BLCA metastasis.

## Author Contributions

ZG and YL: conception and design. HZ, YG, HC, XL, and QG: development of methodology. YL and JH: acquisition of data. ZG, YL, YG, and HZ: analysis and interpretation of data. YL, ZG, H-ML, and XW: writing, review, and/or revision of the manuscript. ZG: study supervision. All authors read and approved the final manuscript.

### Conflict of Interest Statement

The authors declare that the research was conducted in the absence of any commercial or financial relationships that could be construed as a potential conflict of interest.

## Supplementary Material

Figure S1 The Kaplan-Meier curves of OS for 47 stage IV BLCA patients in the unified data of BLCA158 and BLCA57 datasets.

Table S1 Functional analysis for 4061 DEGs (FDR<1%, hypergeometric distribution model).
