# Supplementary figures and images for: A Qualitative Transcriptional Signature for Predicting Recurrence Risk of Stage I–III Bladder Cancer Patients After Surgical Resection
Source: Front Oncol. 2019 Jul 10;9:629. doi: 10.3389/fonc.2019.00629 (PMC6635465; doi:10.3389/fonc.2019.00629)

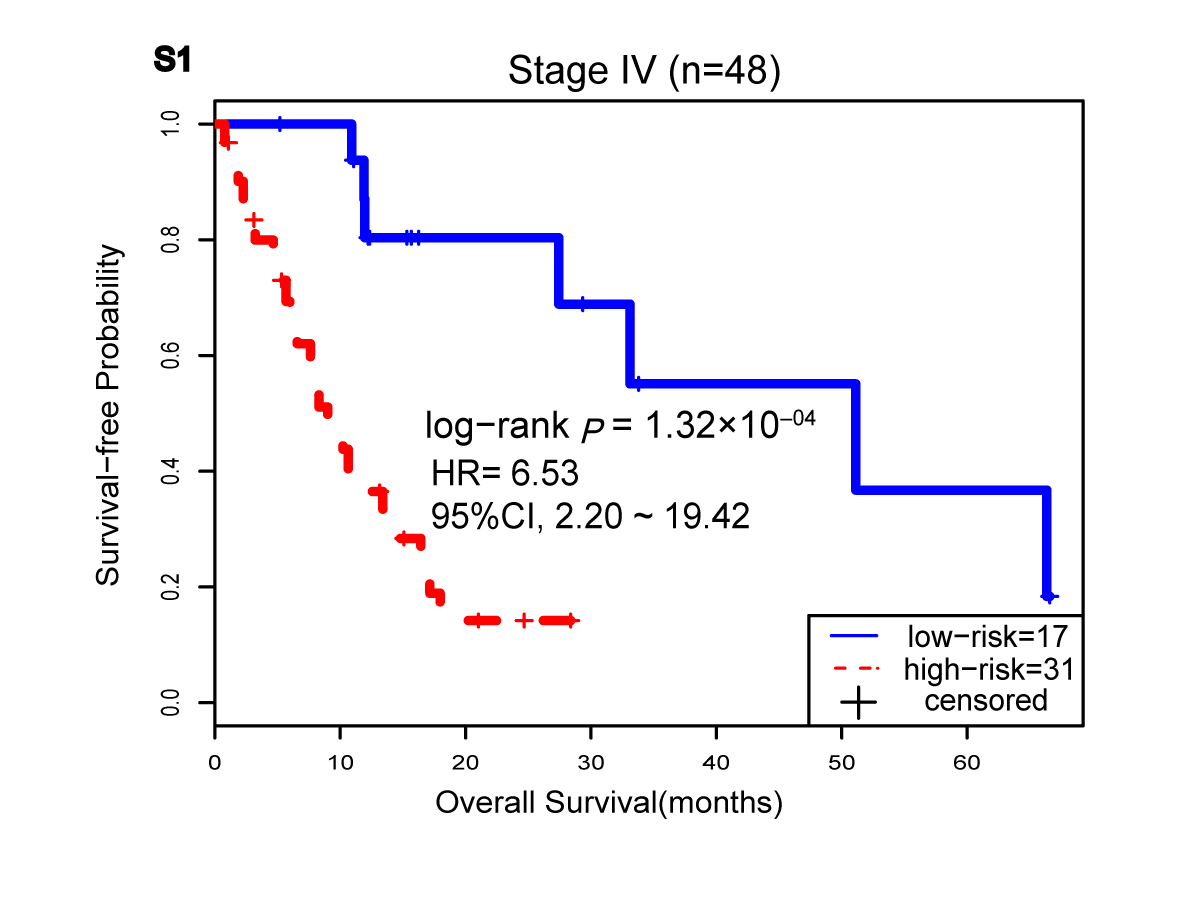

Supplement: Figure S1 — The Kaplan-Meier curves of OS for 47 stage IV BLCA patients in the unified data of BLCA158 and BLCA57 datasets. [file Image_1.TIF]
