# Supplementary material for: A Qualitative Transcriptional Signature for Predicting Recurrence Risk of Stage I–III Bladder Cancer Patients After Surgical Resection
Source: Front Oncol. 2019 Jul 10;9:629. doi: 10.3389/fonc.2019.00629 (PMC6635465; doi:10.3389/fonc.2019.00629)
Supplement: Table S1 — Functional analysis for 4061 DEGs (FDR<1%, hypergeometric distribution model). [file Table_1.DOCX]

**Table S1:** Functional analysis for 4061 DEGs (*FDR*<1%, hypergeometric distribution model)

| goid | name | refnum | interestnum | pvalue | adjustp |
| --- | --- | --- | --- | --- | --- |
| GO:0001501 | skeletal system development | 409 | 165 | 1.88E-06 | 0.000229 |
| GO:0002274 | myeloid leukocyte activation | 131 | 65 | 1.16E-06 | 0.000149 |
| GO:0002443 | leukocyte mediated immunity | 225 | 98 | 5.89E-06 | 0.000663 |
| GO:0002521 | leukocyte differentiation | 384 | 158 | 7.71E-07 | 0.000106 |
| GO:0002576 | platelet degranulation | 87 | 44 | 3.30E-05 | 0.003131 |
| GO:0006955 | immune response | 1186 | 493 | 0 | 0 |
| GO:0007010 | cytoskeleton organization | 939 | 337 | 1.16E-05 | 0.001268 |
| GO:0007155 | cell adhesion | 1393 | 548 | 2.22E-16 | 5.34E-13 |
| GO:0007186 | G-protein coupled receptor signaling pathway | 527 | 202 | 8.47E-06 | 0.000947 |
| GO:0008360 | regulation of cell shape | 117 | 57 | 1.06E-05 | 0.001173 |
| GO:0009888 | tissue development | 1409 | 498 | 6.21E-07 | 8.95E-05 |
| GO:0016477 | cell migration | 1007 | 397 | 3.32E-12 | 3.19E-09 |
| GO:0030154 | cell differentiation | 2788 | 963 | 1.47E-10 | 6.41E-08 |
| GO:0030155 | regulation of cell adhesion | 540 | 230 | 4.96E-11 | 2.56E-08 |
| GO:0030198 | extracellular matrix organization | 285 | 153 | 0 | 0 |
| GO:0031589 | cell-substrate adhesion | 259 | 109 | 1.21E-05 | 0.001308 |
| GO:0032963 | collagen metabolic process | 92 | 57 | 1.26E-10 | 5.69E-08 |
| GO:0043547 | positive regulation of GTPase activity | 507 | 189 | 0.000105 | 0.008898 |
| GO:0051056 | regulation of small GTPase mediated signal transduction | 236 | 98 | 6.10E-05 | 0.005336 |
| GO:0051094 | positive regulation of developmental process | 959 | 361 | 2.16E-08 | 4.59E-06 |
| GO:0051239 | regulation of multicellular organismal process | 2122 | 762 | 5.83E-12 | 4.67E-09 |
| GO:0051301 | cell division | 488 | 187 | 1.86E-05 | 0.001943 |
| GO:0072359 | circulatory system development | 792 | 292 | 4.13E-06 | 0.000483 |
